# Urine-Derived Kidney Progenitor Cells in Cystinosis

**DOI:** 10.3390/cells11071245

**Published:** 2022-04-06

**Authors:** Koenraad Veys, Sante Princiero Berlingerio, Dries David, Tjessa Bondue, Katharina Held, Ahmed Reda, Martijn van den Broek, Koen Theunis, Mirian Janssen, Elisabeth Cornelissen, Joris Vriens, Francesca Diomedi-Camassei, Rik Gijsbers, Lambertus van den Heuvel, Fanny O. Arcolino, Elena Levtchenko

**Affiliations:** 1 Department of Pediatrics, University Hospitals Leuven Campus Gasthuisberg, B-3000 Leuven, Belgium; koenraad.veys@uzleuven.be; 2 Laboratory of Pediatric Nephrology, Department of Development & Regeneration, KU Leuven Campus Gasthuisberg, B-3000 Leuven, Belgium; santeprinciero.berlingerio@kuleuven.be (S.P.B.); tjessa.bondue@kuleuven.be (T.B.); ahmed.reda@kuleuven.be (A.R.); bert.vandenheuvel@med.kuleuven.be (L.v.d.H.); fanny.oliveiraarcolino@kuleuven.be (F.O.A.); 3 Laboratory for Viral Vector Technology and Gene Therapy, Department of Pharmaceutical and Pharmacological Sciences, KU Leuven Campus Gasthuisberg, B-3000 Leuven, Belgium; dries.david@kuleuven.be (D.D.); rik.gijsbers@kuleuven.be (R.G.); 4 Laboratory of Endometrium, Endometriosis & Reproductive Medicine (LEERM), Department of Development & Regeneration, KU Leuven Campus Gasthuisberg, B-3000 Leuven, Belgium; kathi.held@kuleuven.be (K.H.); joris.vriens@kuleuven.be (J.V.); 5 Department of Pathology, Radboud Institute for Molecular Life Sciences, Radboud University Medical Center, 6524 Nijmegen, The Netherlands; martijn.vandenbroek@radboudumc.nl; 6 Department of Pediatrics, Division of Pediatric Nephrology, Amalia Children’s Hospital, Radboud University Medical Center, 6524 Nijmegen, The Netherlands; marlies.cornelissen@radboudumc.nl; 7 Department of Human Genetics, KU Leuven Campus Gasthuisberg, B-3000 Leuven, Belgium; koen.theunis@kuleuven.be; 8 Department of Internal Medicine, Radboud University Medical Center, 6524 Nijmegen, The Netherlands; mirian.janssen@radboudumc.nl; 9 Unit of Pathology, Department of Laboratories, Bambino Gesù Children’s Hospital, IRCCS, 00165 Rome, Italy; francesca.diomedi@opbg.net; 10 Leuven Viral Vector Core, KU Leuven, B-3000 Leuven, Belgium

**Keywords:** cystinosis, kidney progenitors, cell model, gene therapy

## Abstract

Nephropathic cystinosis is an inherited lysosomal storage disorder caused by pathogenic variants in the cystinosin (*CTNS*) gene and is characterized by the excessive shedding of proximal tubular epithelial cells (PTECs) and podocytes into urine, development of the renal Fanconi syndrome and end-stage kidney disease (ESKD). We hypothesized that in compensation for epithelial cell losses, cystinosis kidneys undertake a regenerative effort, and searched for the presence of kidney progenitor cells (KPCs) in the urine of cystinosis patients. Urine was cultured in a specific progenitor medium to isolate undifferentiated cells. Of these, clones were characterized by qPCR, subjected to a differentiation protocol to PTECs and podocytes and assessed by qPCR, Western blot, immunostainings and functional assays. Cystinosis patients voided high numbers of undifferentiated cells in urine, of which various clonal cell lines showed a high capacity for self-renewal and expressed kidney progenitor markers, which therefore were assigned as cystinosis urine-derived KPCs (Cys-uKPCs). Cys-uKPC clones showed the capacity to differentiate between functional PTECs and/or podocytes. Gene addition with wild-type *CTNS* using lentiviral vector technology resulted in significant reductions in cystine levels. We conclude that KPCs present in the urine of cystinosis patients can be isolated, differentiated and complemented with *CTNS* in vitro, serving as a novel tool for disease modeling.

## 1. Introduction

Cystinosis is a rare autosomal recessive lysosomal storage disorder, caused by bi-allelic pathogenic variants in the *CTNS* gene leading to the malfunctioning or absence of the cystine-proton cotransporter cystinosin [1]. It is a multisystem disease in which the kidney is the first and most severely affected organ [2]. In the infantile phenotype, the most common and severe form, a generalized proximal tubular dysfunction (renal Fanconi syndrome) develops in infancy, followed by progressive chronic kidney disease (CKD) leading to end-stage kidney disease (ESKD) [3]. The juvenile phenotype is characterized by a less pronounced Fanconi syndrome and slower kidney function decline. Cysteamine, a cystine-depleting amino-thiol, effectively reduces intracellular cystine accumulation and is currently the only available disease-modifying treatment [3]. Cysteamine treatment has been shown to prolong life expectancy, improve growth, postpone the onset of ESKD and reduce the number of extra-renal manifestations of cystinosis [4,5,6]. However, it offers no cure for renal Fanconi syndrome and does not prevent the need for renal replacement therapy [5,6].

Earlier, we demonstrated a remarkable loss of proximal tubular epithelial cells (PTECs) and podocytes in the urine of cystinosis patients [7]. Therefore, we hypothesized that in compensation for this epithelial cell loss, cystinosis kidneys might show an attempt for regeneration, which could be reflected by the presence of kidney progenitor cells in the urine. The actual existence of a stem/progenitor cell niche in the human adult kidney has been a matter of debate for more than a decade. A majority of studies have suggested the presence of a progenitor cell population by deriving them directly from kidney tissue and characterizing in vitro properties and functional potential in animal models of kidney injury, while few have investigated urine as a source for kidney progenitor cells [8,9,10,11,12,13,14,15,16,17,18,19].

In addition, current cystinosis kidney cell models show several limitations [20]. Primary cells can only be obtained by invasive means via kidney biopsies which are not required for regular clinical care and can be contaminated with several cell types. While these primary cells show a limited proliferative capacity, immortalization might affect the transcriptome, interfere with important cellular pathways and may be unreliable at high passage numbers [20,21,22]. The development of induced pluripotent stem cells (iPSC) is laborious and expensive and iPSC reprogramming might induce genomic side effects [23]. Therefore, alternative cystinosis cell models that demonstrate the primary phenotype of cystinosis and harbor a proliferative capacity without having the disadvantages of immortalization are desirable. These models can further improve our understanding of the cellular pathophysiology of cystinosis and help to elucidate the exclusive functions of cystinosin [20].

In this study, we describe the presence of a niche of kidney progenitor cells in cystinosis patients, which can be non-invasively isolated from urine and serve as a novel tool for disease modeling.

## 2. Materials and Methods

### 2.1. Study Participants

The ethical board of UZ/KU Leuven (Ethische Commissie Onderzoek UZ/KU Leuven) approved the study (s54695) and participants signed an informed consent form. The research was conducted in accordance with the latest version of the Declaration of Helsinki, the principles of Good Clinical Practice (GCP) and all applicable national and international legislation related to research involving human subjects [24].

Fresh urine samples were obtained from non-kidney transplanted nephropathic cystinosis patients, followed at the University Hospital Leuven (UZ Leuven), Belgium and Radboud umc Nijmegen, The Netherlands, and from healthy (not age- or gender-matched) controls. Relevant demographic and clinical data were collected from the medical records of the participants (Table 1).

### 2.2. Estimation of the Number of Undifferentiated Cells in Urine via Quantitative Polymerase Chain Reaction (qPCR)

Vimentin (*VIM*) is a known marker of mesenchymal cells, being expressed by undifferentiated cells of the metanephric mesenchyme in the human fetal kidney. Vimentin-expressing cells have consistently been shown to co-express CD133 and PAX2 in kidney progenitor cells. Therefore, we assessed the presence of undifferentiated cells in the urine of cystinosis patients by quantifying vimentin-positive cells [8,15,25,26,27]. Briefly, a calibration curve for the mRNA expression of vimentin was developed using known numbers of control human kidney stem cells, which express vimentin (kindly provided by Prof. B. Bussolati, Università degli Studi di Torino, Turin, Italy) [8,28] (Figure S1). To establish the calibration curve, cells were sorted by fluorescence activated cell sorting (FACS) using the BD FACSAria III (BD Biosciences, San Jose, CA, USA) at the VIB-KU Leuven FACS Core Facility, in a 96-well plate containing 4 μL of lysis buffer (0.2% TritonX-100 + RNase inhibitor) yielding a range of cells per well from 1 to 500 cells (Figure S1).

The Smart-Seq2 protocol allowed the preparation of cDNA from very low numbers of cells. An adaption of the protocol of Picelli et al. was applied up to PCR purification (step 26), and 18 PCR cycles were performed in step 14. qPCR was performed using the reference gene ß-Actin, and vimentin as a marker for undifferentiated cells [28] (Figure S1). The *VIM* primer used is indicated in Table S4.

After establishing the *VIM* calibration curve, urine samples were collected from cystinosis patients and healthy subjects, centrifuged (200× *g*, 4 °C, 5 min), and the cell pellet was resuspended in phosphate buffered saline (PBS) and diluted 100×. The cycle threshold (ct) value for *VIM* achieved in the qPCR analysis was plotted in the calibration curve for extrapolation of the number of undifferentiated cells voided in urine. Given that cystinosis patients are polyuric, urine samples of cystinosis patients showed a higher volume and were more diluted in comparison with urine samples of healthy control subjects. Therefore, results were normalized to urine volume and creatinine values, the latter to correct for the concentration of the urine sample.

### 2.3. Establishment of Cystinosis Urine-Derived Kidney Progenitor Cells (Cys-uKPCs)

Fresh urine samples were centrifuged (300× *g*, 5 min, room temperature), the supernatant was removed, and the cell pellet was washed with PBS, followed by re-centrifugation. Depending on the size of the cell pellet, cells were seeded in a single or multiple 10 cm Petri dishes and incubated in a medium containing a 1:1 ratio of keratinocyte-serum free medium (Keratinocyte-serum free medium + L-glutamine Gibco^®^ Life Technologies reference number 17005-034, 5 ng/mL epidermal growth factor, 50 ng/mL bovine pituitary extract, 30 ng/mL cholera toxin, 100 U/mL penicillin, 1mg/ml streptomycin) and progenitor cell medium (3/4 Dulbecco’s Modified Eagle’s medium Lonza category number BE12-733F, ¼ Hamm’s F12 Lonza category number BE12615F), 10% fetal bovine serum, 0.4 ug/mL hydrocortisone, 10^−10^ M cholera toxin, 5 ng/mL insulin, 1.8 × 10^−4^ M adenine, 5 ug/mL transferrin, 2 × 10^−9^ M 3,39,5 tri-iodo L-thyronine, 10 ng/mL epidermal growth factor, 1% penicillin-streptomycin) (1:1 mix), in the following, altogether referred to as progenitor medium, at 37 °C in 5% CO_2_ [17,29].

Clonal colonies of cells could be observed after 3–5 days of culturing the fresh urine sample and were picked between 9 and 14 days. Each picked clonal colony is hereafter further named as ‘clone’ [30]. Each clone was seeded in a single well of a 24-well plate (passage number 1, P#1). Upon reaching 70–80% confluence, cells were trypsinized and counted using the BioRad TC20^TM^ automated cell counter (BioRad #145-0101). In passage 2 (P#2), all cells were transferred into a 6 cm cell culture dish, while by the next passage (P#3) 2/3rd of cells were plated in a 10cm dish. From the 4th passage on, a splitting ratio of ¼ was maintained and cells were further proliferated in 10 cm Petri dishes until senescence was reached. Senescence was defined as the inability to form a subconfluent (70–80% confluence) monolayer within 14 days of culture. Mycoplasma tests were performed every 2 months and no bacterial contamination was detected in any of the culture samples.

### 2.4. Characterization of Cys-uKPCs

The isolated clones were characterized as Cys-uKPCs based on their proliferative capacity (doubling time), and by their gene expression profile as assessed by qPCR. The doubling times were calculated according to the following formula: doubling time = duration * log (2)/log (final concentration) − log (initial concentration) [31]. The following kidney progenitor genes were assessed by qPCR: Neural Cell Adhesion Molecule 1 (*NCAM1*), CBP/p300 interacting transactivators with glutamic acid (E)/aspartic acid (D)-rich C-terminal domain (*CITED1*), vimentin (*VIM*) and paired box 2 (*PAX2*); while β-Actin was used as the housekeeping gene. Briefly, mRNA was isolated using the RNeasy Mini or Micro Kit (Qiagen GmbH, Hilden, Germany), according to the manufacturer’s protocol. RNA was synthesized to cDNA using a mix of Oligo (dT) 12–18 Primer, random primers, dNTP mix (100 mM) and SuperScript^TM^ III Reverse Transcriptase, all from Invitrogen. qPCR was executed on a CFX96^TM^ Real-Time PCR Detection System, using Platinum^TM^ SYBR^TM^ Green qPCR SuperMix-UDG w/ROX (Thermo Fisher), 10 μM of primers and 1 μL of cDNA (5 ng/μL). qPCR data were retrieved and processed using the CFX Manager^TM^ software (Bio-Rad, Hercules, CA, USA). All antibodies and primers used are specified in Table S3 and Table S4, respectively. Clones were considered Cys-uKPCs when they (1) showed a high self-renewal capacity (mean doubling time at P#3: 35 h ± 13 h; at P#5: 56 h ± 19 h) that allowed a full characterization of these cells (including gene expression analysis in P#4 and assessment of differentiation to PTEC or podocyte in P#6), (2) cells expressed a panel of kidney progenitor genes *CITED1*, *NCAM1*, *VIM* and *PAX2* at P#4, and (3) showed signs of differentiation to either PTEC or Podocyte as evidenced by either upregulation PTEC- or podocyte-specific genes, respectively, upon subjection to the corresponding differentiation protocol (see below) (Figure S2).

### 2.5. Differentiation of Cys-uKPCs to Podocytes and Proximal Tubular Epithelial Cells

All presumed Cys-uKPC clones were subjected to a differentiation protocol towards podocytes (‘Cys-uKPC-Podo’) in passage #6 via incubation in VRAD medium (DMEM-F12, 10% FBS, 100nM vitamin D3, 100μM all-trans retinoic acid) for 7 days, during which the VRAD medium was refreshed 2 times (every 2–3 days). Expression of podocyte-specific markers synaptopodin (*SYNPO*), podocalyxin (*PODXL*), and Wilms’ Tumor 1 (*WT1*) were assessed by qPCR, and changes in cellular morphology during differentiation were monitored by light microscopy. Depending on gene expression results, also Western blot and immunofluorescence staining were performed.

In parallel, the same Cys-uKPC clones were also subjected to a differentiation protocol towards PTECs (Cys-uKPC-PTEC) in passage #6 by incubation in PTEC medium (DMEM-F12, Insulin 5 μg/mL, Thyroxin 5 μg/mL, Selenium 5 ng/mL, Hydrocortisone 36 ng/mL, EGF 10 ng/mL, Tri-iodothyronine 40 pg/mL, 10% FBS, 1% penicillin-streptomycin) for 7 days, during which the PTEC medium was refreshed 2 times (every 2–3 days). The expression of PTEC-specific markers aquaporin 1 (*AQP1*) and P-glycoprotein (P-gp) (*ABCB1)* was demonstrated by rt-PCR and qPCR. Depending on the gene expression results, a Western blot was performed.

### 2.6. Western Blot

Cell pellets were lysed in RIPA lysis buffer (Thermo Fisher Scientific, Waltham, MA, USA) supplemented with protease and phosphatase inhibitors. Total protein concentrations were quantified using a Pierce BCA Protein Assay Kit (Thermo Fisher Scientific, Waltham, MA, USA). For the synaptopodin, podocalyxin and aquaporin 1 Western blot, 10 μg total protein were loaded, while for the Cystinosin-3HA Western blot, 15 μg total protein was loaded on a precast gel NuPAGE Novex 4–12% Bis–Tris (Thermo Fisher Scientific, Waltham, MA, USA) and transferred on a nitrocellulose membrane using an iBlot2 dry blotting system.

Membranes were incubated overnight at 4 °C with primary antibodies against aquaporin 1, synaptopodin, podocalyxin and β-Actin on a blocking buffer. Next, membranes were incubated with HRP-linked secondary antibodies anti-rabbit or mouse in a 1:2000 dilution in 5% milk in TBS-T. Proteins were visualized using ECL Substrate (Thermo Fisher Scientific, Waltham, MA, USA). Images were acquired using the Syngene Chemi XRQ System and quantified with ImageJ software.

The specification of all antibodies is described in Table S3.

### 2.7. Immunofluorescence Staining and Microscopy

Twenty to forty thousand cells were seeded, fixed, and permeabilized, followed by blocking and incubation of primary and secondary antibodies (Table S3). Microscopy was performed on the Nikon Eclipse Ci microscope (Nikon Corporation, Tokyo, Japan), while confocal microscopy images were recorded on the Zeiss^®^ LSM 880—Airyscan (Carl Zeiss Microscopy GmbH, Jena, Germany) (Cell and Tissue Imaging cluster/Cell Imaging Core (CIC), Pieter Vanden Berghe, KU Leuven, B-3000 Leuven, Belgium). Microscopy images were processed and analyzed using the Zeiss ZEN Black and Blue imaging software and Fiji/ImageJ.

### 2.8. Functional Assessment of Cys-uKPC-Podo and Cys-uKPC-PTEC

#### 2.8.1. Albumin Endocytosis Assay

The functionality of Cys-uKPC-Podo was analyzed by monitoring the capacity of endocytic uptake of albumin in an Alexa Fluor^TM^ 555 albumin endocytosis assay in passage #7 (Figure S2). Eighty thousand Cys-uKPCs, Cys-uKPC–Podo and cystinosis conditionally immortalized podocytes (ciPodocyte^CTNS−/−^) were seeded on glass coverslips. Following differentiation, cells were starved by incubation in serum- and supplement-free medium (DMEM-F12; Lonza) for 2 h. Hereafter, incubation with a complete medium supplemented with 100 μg/mL of Alexa Fluor^TM^ 555 conjugated bovine serum albumin (BSA) (Life Technologies, Carlsbad, CA, USA) was performed for 60 minutes in parallel at 37 °C and at 4 °C, the latter for confirming the temperature dependency of specific receptor-mediated albumin endocytosis in podocytes and to rule out non-specific binding and uptake of albumin [32]. The uptake of the labeled BSA was analyzed by fluorescence microscopy using the Nikon Eclipse Ci microscope (Nikon Corporation, Tokyo, Japan). Quantification of BSA uptake was analyzed using Fiji/ImageJ software, and based on the principle of corrected total cell fluorescence (CTCF) [33]. A correct interpretation of actual BSA uptake by the individual cells was ensured by visualization of the cell border via enhancement of image brightness and contrast in Fiji/ImageJ. The experiment was performed in one Cys-uKPC clone differentiated (in triplicate) to Cys-uKPC-Podo (Cys-uKPC #1-Podo) and an average of 5 images containing on average 23 cells per image were quantified per condition.

#### 2.8.2. Calcium Influx Assay

Given the pivotal importance of calcium dynamics in podocytes, Cys-uKPC-Podo should demonstrate a more mature system of calcium signaling as evidenced by higher responsiveness to agonists of transient receptor potential calcium channels (TRPCs) [34,35,36]. TRPC3 is known to be widely expressed in renal epithelial cells, while TRPC6 is of specific importance in podocytes [37,38]. Therefore, the functionality of Cys-uKPC-Podo was also analyzed by evaluating the calcium influx in a Fura calcium imaging assay in comparison to their undifferentiated Cys-uKPC counterparts in passage #7 [17,39]. Cys-uKPCs were seeded on glass coverslips and were differentiated to podocytes (Cys-uKPC-Podo). Cys-uKPC-Podo was incubated with 2 μM Fura-2 acetoxymethyl ester (Invitrogen^TM^) for 20 min at 37 °C. Standard imaging solutions consisted of 150 mM NaCl, 2 mM CaCl_2_, 1 mM MgCl_2_, 10 mM HEPES (pH 7.4 by NaOH). Perfusion of the bath solutions was based on gravity via a multi-barreled pipette tip with a single outlet of 0.8 mm diameter. Intracellular Ca^2+^ concentration was determined based on the ratio of fluorescence detected upon alternating excitation at 340 and 380 nm, using a Lambda XL illuminator (Sutter Instruments, Novato, CA, USA) and an Orca Flash 4.0 camera (Hamamatsu Photonics Belgium, Mont-Saint-Guibert, Belgium) on a Nikon Eclipse Ti fluorescence microscope (Nikon Benelux, Brussels, Belgium). Data were analyzed using NIS-Elements software (Nikon) and IgorPro 6.2 (WaveMetrics, Portland, OR, USA). 1-oleoyl-2-acetyl-sn-glycerol (OAG) (Sigma-Aldrich, Burlington, MA, USA), a TRPC6 agonist, was applied (150 mM) in order to stimulate calcium influx and assess the response rate of the cell type studied. Cells showing an increase in calcium amplitude higher than 50 nM and a slope increase higher than three times the standard deviation were considered as responders to OAG stimulation. Only cells that responded to the positive control, ionomycin, at the end of the experiment were analyzed. All experiments were performed on at least 3 independent coverslips. Undifferentiated Cys-uKPCs were also assessed, while wild-type ciPodocytes (ciPodocyte^WT^), incubated for 10–14 days at 37 °C, were used as a positive control.

#### 2.8.3. Transferrin Endocytosis Assay

The functionality of Cys-uKPC-PTEC with regard to receptor-mediated endocytosis was assessed via a fluorescence-labeled transferrin endocytosis assay in passage #7. Cys-uKPCs were differentiated to PTEC (Cys-uKPC-PTEC), as previously described, and starved for 60 min at 37 °C in basal medium (DMEM F-12 with 1% BSA). Thereafter, Cys-uKPC-PTEC were washed with ice-cold PBS and incubated with an Alexa Fluor^TM^ 555 conjugated transferrin (TF555, Life Technologies) (25 μg/mL) in basal medium for 30 min at 4 °C, followed by incubation at 37 °C for 15, 30, 45 and 60 min in complete medium. Immunofluorescence imaging was performed using the Eclipse CI microscope (Nikon, Tokyo, Japan). For analysis, 30 cells were studied per time point, using the ImageJ software. The fluorescence per cell was quantified and the background signal subtracted. For the analysis of endocytic uptake at different time points, data were normalized to time point zero.

### 2.9. Kidney Biopsy Specimen & Immunohistochemistry

A kidney tissue specimen was retrieved from the native kidney of one nephropathic cystinosis patient who had undergone nephrectomy because of ongoing renal Fanconi syndrome following kidney transplantation and assessed for the presence of the kidney progenitor cells via staining of CD133 and PAX2. Since this specimen was retrieved for clinical purposes, retrospective coverage by an ethical board for approval was not applicable. The clinical characteristics of the patient are presented in Table S3. Stainings on control biopsies are shown in Figure S5. The PAX2 control staining was performed in a developing kidney specimen, more specifically, from a healthy fetus of a gestational age of 28 weeks, of whom a biopsy was taken for regular clinical care purposes. The CD133 control staining was performed in the adult transplant kidney of a healthy 13-year-old teenager, while a kidney biopsy in the context of acute tubular injury, taken for regular clinical care purposes, was used as a positive control for CD133. The specification of the antibodies that were used for this application is indicated in Table S3.

### 2.10. Lentiviral Vector Design and Transduction Experiments

The Leuven Viral Vector Core (LVVC) developed several self-inactivating (SIN) lentiviral vector (LV) constructs, using a human elongation factor-1 alpha (EF-1 α) promoter to drive the expression of either *CTNS* (full-length cDNA; tagged with a 3HA tag at its C-terminus) or *eGFP* or *dATP13A2* (a deactivated version of ATP13A2, a lysosomal transmembrane protein) as a transgene, followed by an EMCV IRES-puromycin antibiotic resistance cassette, referred to as LV_CTNS-3HA, LV_eGFP and LV_dATP13A2, respectively (Figure S7, panel A) [40]. Validation of the LV constructs was performed in skin fibroblasts, that were obtained from a cystinosis patient prior to cysteamine treatment, via immunofluorescence staining and Western blot analysis (Figure S7, panel B–E) [41]. Two Cys-uKPC clonal cell lines (Cys-uKPC #1, Cys-uKPC #7) were transduced, resulting in the following cell lines: Cys-uKPC #1 LV_CTNS-3HA and Cys-uKPC #7 LV_CTNS-3HA, Cys-uKPC #1 LV_eGFP and Cys-uKPC #7 LV_eGFP, and Cys-uKPC #7 LV_dATP13A2. In order to ensure a single integrated viral vector copy per cell, viral vector transduction was conducted employing a limiting dilution series. Cells were transduced for 72 h and subsequently selected by adding puromycin (1 μg/mL) to the medium. We selected the highest dilution that still resulted in surviving cells upon puromycin selection (<20% surviving cells, which indicates a single integrated viral vector copy, corresponding to an MOI < 0.5) [42]. The corresponding non-functional p24 titers for this condition were, for Cys-uKPC #1 LV_CTNS-3HA 0.003 pg p24/mL, in Cys-uKPC #1 LV_eGFP 0.0009 pg p24/mL, while in Cys-uKPC #7 LV_CTNS-3HA the p24 titer was 0.06 pg p24/mL, 0.02 pg p24/mL for the Cys-uKPC #7 LV_eGFP and 0.04 pg p24/mL for the Cys-uKPC #7 LV_dATP13A2 transduced conditions. The resulting selected transduced Cys-uKPCs were harvested for mRNA isolation and assessment of intracellular cystine levels.

### 2.11. Cystine Measurements

One million cells were washed with PBS, 200 μL of ice-cold N-ethylmaleimide (‘NEM’) was added to block free thiol groups, followed by collecting the cells by scraping. An amount of 100 μL of 12% sulfosalicylic acid (‘SSA’) was added for protein precipitation, vortexed for 30 s for homogenization, and centrifuged (10 min, 10,000× *g*, 4 °C). The cystine-containing supernatants were isolated for the cystine assay and kept immediately at –80 °C, while the protein pellet was incubated with 300 μL of 0.1M NaOH (Sigma-Aldrich) overnight, and then transferred to –80 °C until the total protein concentration was determined by a BCA assay. Cystine was measured by liquid chromatography coupled to tandem mass spectrometry (‘LC-MS/MS’) in a kind collaboration with the Laboratory of Pathology and Metabolism of the Bambino Gesù Pediatric Hospital, Rome. Cystine concentrations were expressed as nmol cystine/mg protein.

### 2.12. Statistical Analysis

Graphpad Prism (version 9.3.1 (350) for Mac OS X) (GraphPad Software, La Jolla, CA, USA, www.Graphpad.com, accessed on 7 December 2021) was used for the statistical analysis. The D’Agostino & Pearson normality test was applied in order to check for normality of the distribution of the medians of the parameters of the cystinosis patients and the control subjects. Depending on this distribution, a two-tailed unpaired Student’s *t*-test or Mann–Whitney U test was performed.

## 3. Results

### 3.1. Nephropathic Cystinosis Is Characterized by a Loss of Undifferentiated Cells in Urine

To test our hypothesis that a significant loss of kidney epithelial cells in cystinosis patients could be compensated with an attempt for regeneration, we first explored whether undifferentiated cells, characterized by expression of vimentin, are present in the urine of cystinosis patients [7].

Therefore, we adapted a method used for single-cell RNA extraction and amplification to quantify vimentin-expressing cells voided in urine (Figure S1). Urine samples were collected from nine cystinosis patients and nine healthy subjects (Table S1).

**Figure 1:**
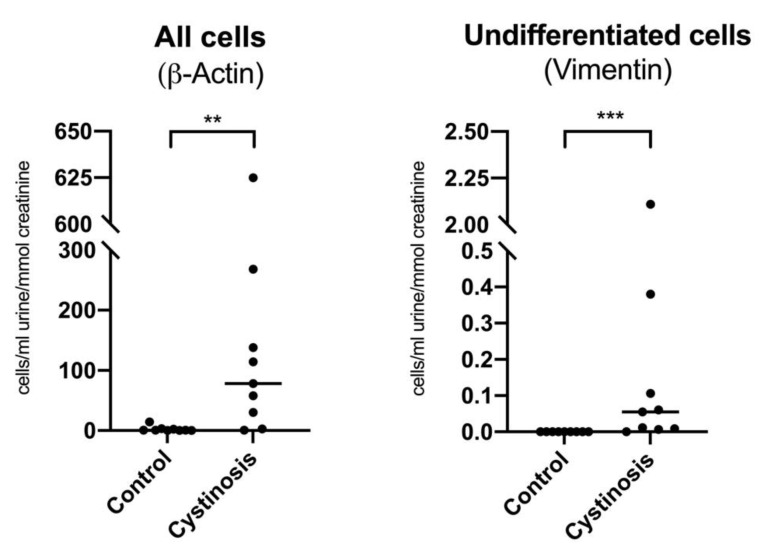
Quantification of undifferentiated cells in urine of cystinosis patients versus healthy controls. Total number of cells (β-Actin signal, left) and number of undifferentiated cells (vimentin signal, right) voided in urine of cystinosis patients (*n* = 9) compared to healthy controls (*n* = 9). The cycle threshold (ct) value achieved in the qPCR analysis was plotted in a calibration curve for estimation of the total number of cells and the number of undifferentiated cells voided in urine, which was normalized to urine volume and urine creatinine values. Statistical significance was evaluated using Mann–Whitney U test comparing controls versus cystinosis patients; median is indicated with the horizontal line in the midst of the individual values. *p* < 0.01: **; *p* < 0.001: ***.

Cystinosis patients presented a higher number of undifferentiated cells in the urine, which were not observed in healthy subjects (Figure 1). There was no correlation between the number of undifferentiated cells with age (Spearman r −0.25, *p =* 0.52), proteinuria (Spearman r −0.62, *p* = 0.09), kidney function (eGFR; Spearman r 0.22, *p* = 0.58), the white blood cell cystine level (WBC cystine level) (Spearman r 0.15, *p* = 0.71), or the cystinosis genotype (Figure S3).

### 3.2. Undifferentiated Cells in Urine of Cystinosis Patients Comprise Kidney Progenitor Cells (Cys-uKPCs)

In order to explore whether these undifferentiated cells comply as kidney progenitor cells, we isolated, expanded and characterized clonal cell lines following a standardized protocol in line with our method on the isolation and expansion of kidney progenitor cells from the urine of preterm born neonates (neonatal kidney stem progenitor cells, nKSPCs) (Figure S2) [17].

Therefore, urine samples of all recruited cystinosis patients were cultured in a progenitor medium (Table 1; Figure S2).

**Figure 2:**
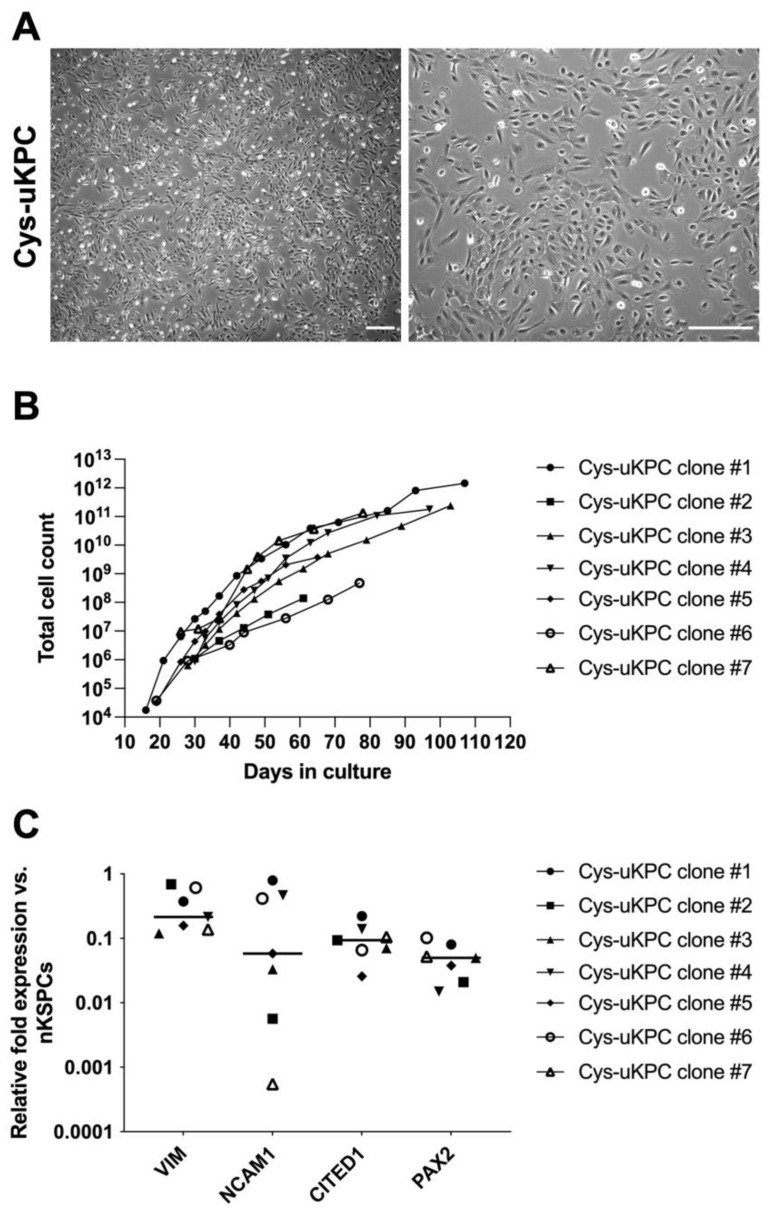
Characterization of cystinosis urine-derived kidney progenitor cell (Cys-uKPC) clones. (**A**) Phase contrast microscopy of a representative clone of Cys-uKPCs (Cys-uKPC #1) shows the characteristic petal-like pattern of in vitro expansion and organization (left panel) and the spindle-shaped to rhombic cellular morphology (right panel). Scale bar: 250 μm; (**B**) In vitro proliferative capacity of the seven established Cys-uKPC clones. Total number of cells grown versus days in culture before Cys-uKPC clones showed senescence and stopped proliferating. Each specific Cys-uKPC clone is represented by a symbol that is similarly used in panel (**B**,**C**) (see the legend on right in the figure). (**C**) Relative fold expression of a panel of selected nephrogenesis genes for characterizing the Cys-uKPC clones (*n* = 7), relative to β-actin and normalized to the expression in human neonatal kidney stem/progenitor cells (nKSPCs). In the midst of the individual values, the median of the values is represented by the horizontal line.

This culture yielded an average of 0.9 clonal colonies/ml urine volume (SD 1.7 clonal colonies/ml urine volume). The clonal colonies that showed the highest capacity for self-renewal were picked, expanded and further referred to as ‘clones’ (Figure S2). These clones showed a spindle-shaped morphology and a characteristic petal-like pattern of organization, resembling that of kidney stem progenitor cells isolated from the urine of preterm neonates (Figure 2A) [17].

Since patient #6 and patient #9 showed a remarkable higher yield in a number of clonal colonies growing in the progenitor medium compared with other patients (Table 1) and both represent the full clinical spectrum of nephropathic cystinosis (patient #9: infantile phenotype, patient #6: juvenile phenotype), clonal colonies from only these two patients were selected for further experiments.

Seven clones, assigned as Cys-uKPC #1 until #7, that were isolated from these two patients (patient #6: Cys-uKPC clone #1–5; patient #9: Cys-uKPC clone #6 & #7), stayed in culture for an average 86 ± 18 days and on average 11 ± 3 passages before reaching senescence. The doubling times observed per clone in the earliest passage, in which the automated counting of cells was possible, ranged from 20.87 to 50.58 hours. The total number of cells yielded per clone before reaching senescence ranged from 1.39 × 10^8^ to 1.46 × 10^12^ (Figure 2B). All seven clones showed expression of kidney progenitor genes *NCAM1*, *CITED1*, *VIM* and *PAX2* (Figure 2C) as assessed by qPCR in passage #4, in which nKSPCs were applied as a positive control.

As a result, these seven clones were used for assessing their differentiation potential into kidney epithelial cells and were presumed to be cystinosis urine-derived kidney progenitor cell (Cys-uKPC) clones.

### 3.3. Differentiation of Cys-uKPCs In Vitro towards Functional Podocytes and PTECs

#### 3.3.1. Differentiation of Cys-uKPCs into Functional Podocytes

**Figure 3:**
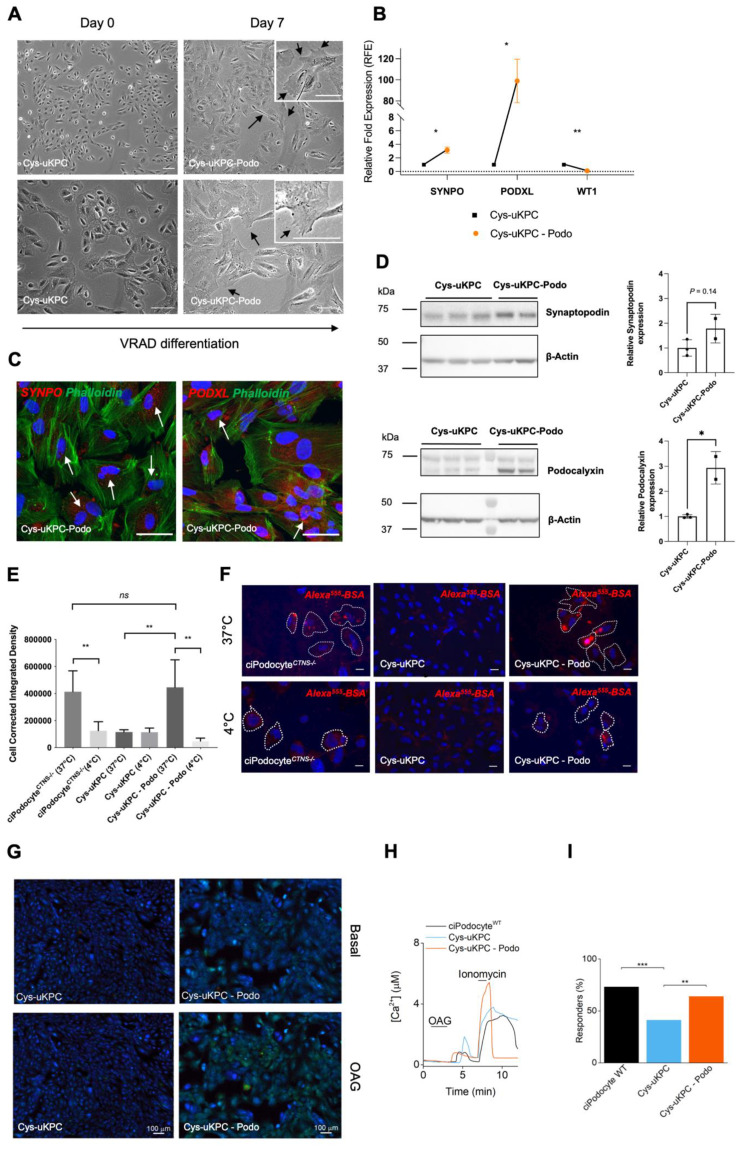
Differentiation of Cys-uKPC in vitro into functional podocytes. (**A**) Phase contrast microscopy of a Cys-uKPC clone with a podocyte fate (Cys-uKPC #1), demonstrating the cellular morphological changes while undergoing differentiation from kidney progenitors (Cys-uKPC; day 0, left column) towards podocytes (Cys-uKPC-Podocyte; day 7, right column) via incubation in VRAD medium. During differentiation, cells become enlarged and show cellular protrusions (black arrows), which are specifically depicted in the zoomed subpanels in the right upper corners of the microscopy pictures in the right column. A proportion of cells became bi-or multinucleated, which can be better appreciated in panel C (white arrows). Similar results were obtained for the other clones. While the lower magnification pictures (top panels) demonstrate the growth pattern and organization of cells, the higher magnification pictures (bottom panels) illustrate better cellular morphology. Scale bar: 100 μm; (**B**) qPCR analysis of podocyte-specific gene expression in Cys-uKPC-derived podocytes relative to β-actin and normalized to Cys-uKPCs of a Cys-uKPC clone (Cys-uKPC #1) with a podocyte fate. Cys-uKPC-Podo data is indicated with an orange dot which represents the mean with bars representing the SEM of three independent experiments with three technical replicates each; Cys-uKPC data is indicated with a black square. *p* < 0.05: *; (**C**) Immunofluorescence staining for podocyte-specific proteins synaptopodin and podocalyxin (red), and F-actin filament cytoskeletal staining via phalloidin (green) in Cys-uKPC-Podo. Nuclei are stained using DAPI. Bi- or multinucleated cells are indicated with white arrows. Scale bar 50 μm. Results depicted originate from the Cys-uKPC clone #1; the individual color channels of each merged immunofluorescence picture depicted here can be appreciated in Figure S4; (**D**) Western blot and quantification for podocyte-specific proteins synaptopodin and podocalyxin in Cys-uKPC-Podo compared to their undifferentiated kidney progenitor counterparts (Cys-uKPC). Increased expression of synaptopodin and podocalyxin is observed in the Cys-uKPC-Podo condition compared to Cys-uKPC, with the corresponding quantification depicted on the right. Results depicted originate from the Cys-uKPC clone #1; (**E**) Cys-uKPC-Podo show a significantly increased capacity for albumin endocytosis that is comparable to that observed in ciPodocyte^CTNS −/−^, in contrast to their undifferentiated Cys-uKPCs counterparts. Quantification of the albumin endocytosis assay using cell corrected integrated density of the signal originating from Alexa Fluor^TM^ 555 labeled albumin in the various conditions: 118,172 AU vs. 402,791 AU; actual difference: 284,619 AU; 95% CI of difference: 211,091 to 454,293; *p* = 0.003. Cell corrected integrated density ciPodocyte^CTNS−/−^ vs. Cys-uKPC-Podo: 442,469 AU vs. 402,791 AU; actual difference: −39,678 AU; 95% CI of difference: −216,228 to 269,379, *p* > 0.99; (**F**) Representative immunofluorescent images of the Alexa Fluor^TM^ 555 albumin endocytosis assay for ciPodocyte^CTNS−/−^, Cys-uKPC and Cys-uKPC-Podo at 37 °C and 4 °C. Results depicted originate from the Cys-uKPC clone #1. The dotted line identifies the border of the individual cells. (**G**) Cys-uKPC-Podo shows an increased number of responders to OAG in a Fura-2AM calcium influx assay compared to their undifferentiated Cys-uKPC counterparts. Cys-uKPC-Podo responders resemble the number of responders in ciPodocytes^WT^. Results depicted originate from Cys-uKPC clone #1; microscopy fluorescence images from Cys-uKPC and Cys-uKPC-Podo during basal conditions and in presence of OAG (150 µM). Low to high calcium is indicated on a color scale that ranges from blue (low calcium) over green (medium calcium) to red (high calcium); (**H**) Example calcium traces of one cell recorded from ciPodocytes*^CTNS^*^WT^ (black), Cys-uKPC (blue) and Cys-uKPC-Podo (orange) during a standard Fura-2AM calcium imaging protocol. Percentage of responders in ciPodocytes^WT^ (total of 221 cells), Cys-uKPC (total of 440 cells) and Cys-uKPC-Podo (total of 278 cells); (**I**) Statistical significance between all groups were tested with a Chi-Square test (*p* = 2.8 × 10^−5^). *p* < 0.05: *; *p* < 0.01: **; *p* < 0.001: *** corrected with the Bonferroni correction for multiple-comparison of *n* = 3 groups.

All seven Cys-uKPC clones (#1–#7) were subjected to the podocyte differentiation protocol using the VRAD medium. Following differentiation, in three of these seven Cys-uKPC clones (Cys-uKPC #1, #5, #6), the cell size increased, and cells showed multiple cellular protrusions (Figure 3A), while a proportion of cells became bi-or multi-nucleated (Figure 3C). At the mRNA level, a significant upregulation of podocyte-specific genes *SYNPO* and *PODXL* was observed (Figure 3, panel B). These differentiated Cys-uKPC clones were hereafter coined Cys-uKPC-Podo. Although Cys-uKPC-Podocytes showed a significantly lower expression of WT1 in comparison to Cys-uKPCs, kidney progenitor cells are known to show a high expression of WT1 [43]. Therefore, downregulation of WT1 following differentiation of kidney progenitors towards podocytes should not be regarded as a sign of unsuccessful differentiation [43].

We further explored protein expression of podocyte-specific genes and podocyte-related functionality in one of these three Cys-uKPC-Podo cell lines (Cys-uKPC #1-Podo), compared to its undifferentiated counterpart (Cys-uKPC #1). At the protein level, increased expression of synaptopodin and podocalyxin in Cys-uKPC-Podo compared to Cys-uKPCs was confirmed and consistent with the results of the relative increase in *SYNPO* and *PODXL* gene expression data (Figure 3C,D).

Given that the endocytic process in podocytes is fundamental to maintaining the glomerular filtration barrier, we assessed the endocytosis capacity in a fluorescence-labeled albumin endocytosis assay [44,45]. We demonstrated that, while no uptake of albumin was observed in the undifferentiated Cys-uKPC (Cys-uKPC #1), effective endocytosis of albumin was present in Cys-uKPC-Podo (Cys-uKPC #1-Podo; Figure 3E,F), comparable with a conditionally immortalized cystinosis podocyte (ciPodocyte*^CTNS^*^−/−^). By comparing a 37 °C and 4 °C condition, we confirmed the temperature dependency of specific receptor-mediated albumin endocytosis in podocytes and thus were able to rule out non-specific binding and uptake of albumin. In addition, several studies demonstrated the vital role of Ca^2+^ signaling in podocytes [34,35,36,37,39,46,47]. Therefore, we evaluated the potential of calcium uptake and demonstrated that Cys-uKPC-Podo (Cys-uKPC #1-Podo) show an increased number of responders to the TRPC6 agonist OAG in a Fura-2AM calcium influx assay, compared to Cys-uKPCs (Cys-uKPC #1; Figure 3G–I). The percentage of OAG-responders from Cys-uKPC–Podo was comparable to wild-type ciPodocytes (ciPodocyte*^CTNS^* ^WT^) (Figure 3I).

#### 3.3.2. Differentiation of Cys-uKPCs into Functional PTECs

**Figure 4:**
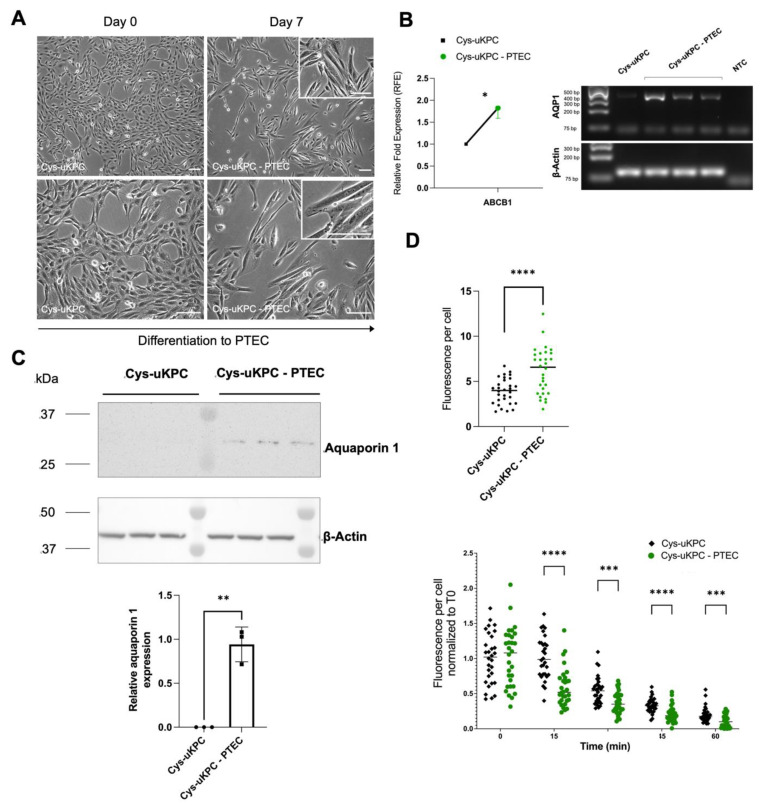
Cys-uKPCs can differentiate in vitro into functional proximal tubular epithelial cells. (**A**) Phase contrast microscopy of a Cys-uKPC clone with a PTEC fate, demonstrating the cellular morphological changes during differentiation (day 0, left column) towards a PTEC (day 7, right column), which is depicted more in specific in the zoomed subpanels in the right upper corner of the microscopy pictures in the right column. Scale bar: 100 μm. Results depicted originate from Cys-uKPC clone #7; (**B**) (**left**) qPCR analysis of PTEC-specific gene expression in Cys-uKPC-PTEC relative to β-actin and normalized to Cys-uKPCs of a Cys-uKPC clone (Cys-uKPC #7) with a PTEC fate. Cys-uKPC-PTEC data is indicated with a green dot, which represents the mean with bars representing the SEM of three independent experiments with three technical replicates each; Cys-uKPC data is indicated with a black square. *p* < 0.05: *; (**right**) rt-PCR analysis of *AQP1* gene expression in Cys-uKPC-PTEC relative to β-actin, in comparison with Cys-uKPC and negative template control (NTC) showing exclusive gene expression of *AQP1* in Cys-uKPC-PTECs in contrast to Cys-uKPCs (**C**) Western blot analysis and quantification of aquaporin 1 expression in one Cys-uKPC-PTEC clone. Aquaporin 1 is undetectable in the undifferentiated Cys-uKPC, while it is clearly expressed in the Cys-uKPCs-PTEC in all three independent differentiation experiments (*p* = 0.0012: **; mean ± SD Cys-uKPC 0.00 ± 0.00; Cys-uKPC-PTEC 0.94 ± 0.2). Results depicted originate from Cys-uKPC clone #7; (**D**) Receptor-mediated endocytosis of transferrin is enhanced in Cys-uKPC-PTEC as observed in an Alexa Fluor^TM^ 555 labeled transferrin endocytosis assay. Binding of transferrin is significantly higher in Cys-uKPC-PTEC compared to Cys-uKPCs, as observed as the fluorescence per cell of Alexa Fluor^TM^ 555 labeled transferrin at time point 0 in the Alexa Fluor^TM^ 555 labeled transferrin endocytosis assay (upper part of panel, right graph). Statistical significance was reached in an unpaired *t*-test (*p* < 0.0001: ****; mean ± SD of fluorescence per cell in Cys-uKPC 3906 ± 1415, Cys-uKPC-PTEC 6178 ± 2547). Endocytic processing of transferrin is significantly enhanced in Cys-uKPC-PTECs compared to Cys-uKPCs as observed as the fluorescence per cell normalized to time point 0 at several time points (15, 30, 45 and 60 min) following internalization of transferrin via receptor-mediated endocytosis (lower part of the panel). Statistical significance was reached for time points 15, 30, 45 and 60 min via a Mann–Whitney test (*p* < 0.0001: ****, *p* < 0.001: ***; median (IQR)) of fluorescence per cell normalized to time point 0 in Cys-uKPC time point 15 min 0.99 (0.77; 1.22) compared to Cys-uKPC-PTEC time point 15 min 0.5191 (0.39; 0.72), in Cys-uKPC time point 30 min 0.54 (0.38; 0.64) compared to Cys-uKPC-PTEC time point 30 min 0.35 (0.25; 0.48), in Cys-uKPC time point 45 min 0.33 (0.26; 0.41) compared to Cys-uKPC-PTEC time point 45 min 0.19 (0.13; 0.27), and Cys-uKPC time point 60 min 0.18 (0.14; 0.23) compared to Cys-uKPC-PTEC time point 60 min 0.01 (0.03; 0.17).

All seven Cys-uKPC clones (#1–#7) were subjected to the PTEC differentiation protocol. Following differentiation, alterations in cellular morphology and upregulation of PTEC-specific genes were observed in six of these seven Cys-uKPC clones (Cys-uKPC #2–5 & #7), hereafter coined Cys-uKPC-PTEC. These alterations at the cellular level comprise cellular enlargement and the acquisition of an elongated, spindle- to tubular-like shape (Figure 4A), along with the significant upregulation of PTEC-specific genes *ABCB1*, and *de novo* expression of *AQP1* at the mRNA level (Figure 4B). We further explored the protein expression of PTEC-specific genes and PTEC-related functionality in one of these Cys-uKPC-PTEC cell lines (Cys-uKPC #7-PTEC), compared to its undifferentiated counterpart (Cys-uKPC #7). Protein expression of aquaporin 1 was confirmed exclusively in Cys-uKPC-PTEC in comparison with the undifferentiated Cys-uKPC (Figure 4C).

We assessed the functionality of Cys-uKPC-PTEC by evaluating receptor-mediated endocytosis of transferrin and demonstrated a significant increase in the binding of fluorescent transferrin at time point 0 in the Cys-uKPC-PTEC (Cys-uKPC #7-PTEC) compared with the undifferentiated Cys-uKPC (Cys-uKPC #7), which suggests an increased expression of endocytosis receptors at the cell surface, a feature of mature PTECs (Figure 4D, upper graphs). In addition, endocytic trafficking of transferrin in Cys-uKPC-PTECs (Cys-uKPC #7-PTEC) was more enhanced in comparison to the undifferentiated Cys-uKPC (Cys-uKPC #7; Figure 4D, lower graph).

Taken together, we demonstrated that KPCs are voided in the urine of cystinosis patients, are clonogenic, can proliferate exponentially in vitro, and can be differentiated towards functional specific cells of the nephron epithelium.

### 3.4. A kidney Progenitor Cell Niche Is Present In Situ in the Kidneys of a Nephropathic Cystinosis Patient

The presence of Cys-uKPCs in the urine of cystinosis patients suggests an attempt at kidney regeneration. Therefore, we sought to demonstrate the presence of these cells in the native kidney of cystinosis patients. As previous studies characterized kidney progenitor cells as being PAX2^+^/CD133^+^, we performed immunochemistry on the kidney tissue of one nephropathic cystinosis patient by use of these markers [8,15,25,26,27]. The clinical characteristics of the patient are described in Table S2. PAX2 CD133 double-positive cells were present in the patient’s tissue scattered throughout the parietal epithelium of the Bowman’s capsule and the tubules (Figure S6, panel A, B).

### 3.5. Reduction in Cystine Levels in Cys-uKPCs via an Ex Vivo Gene Addition Approach

We aimed to explore the feasibility of ex vivo *CTNS* cDNA gene addition via lentiviral vector (LV) technology, in which Cys-uKPCs could be used as a tool for disease modeling.

First, we validated the LV constructs (LV_CTNS-3HA) in cystinosis fibroblasts, confirming CTNS protein expression via immunofluorescent staining and Western blot analysis (Figure S7, panel B–E).

**Figure 5:**
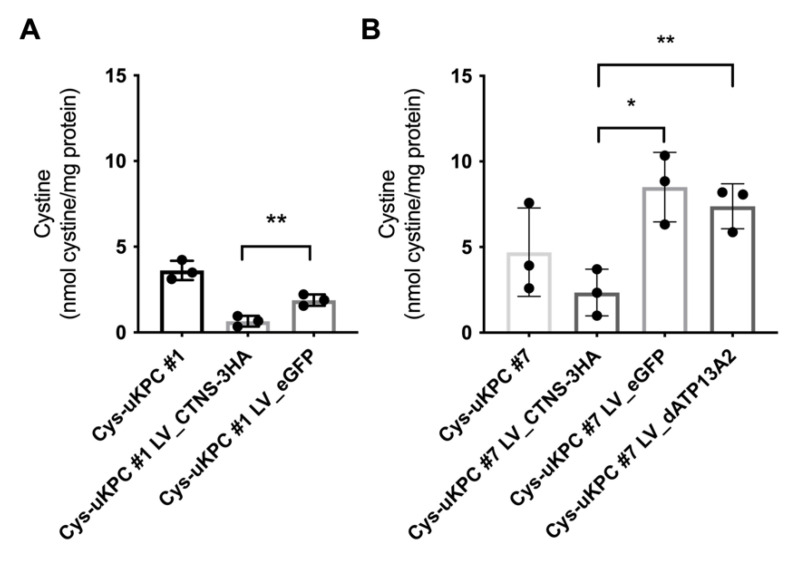
Complementation of *CTNS* via lentiviral vector transduction in two Cys-uKPC clonal cell lines significantly reduces intracellular cystine levels. Cystine measurement by HPLC in Cys-uKPCs (Cys-uKPC #1, #7), Cys-uKPCs transduced with LV_CTNS-3HA (Cys-uKPC #1 LV_CTNS-3HA & #7 LV_CTNS-3HA) and Cys-uKPCs transduced with LV_eGFP (Cys-uKPC #1 LV_eGFP and #7 LV_eGFP) and/or LV_dATP13A2 (Cys-uKPC #7 LV_dATP13A2) as vehicle controls. The graph shows mean ± SD of three independent measurements; *p* < 0.05: *; *p* < 0.01: **. (**A**) The cystine level of Cys-uKPC #1 LV_CTNS-3HA (0.66 ± 0.31 nmol cystine/mg protein) is significantly lower compared to the eGFP-transduced Cys-uKPC #1 as vehicle control (Cys-uKPC #1 LV_eGFP: 1.89 ± 0.34; difference between means of Cys-uKPC #1 LV_CTNS-3HA and Cys-uKPC #1 LV_eGFP cystine level: 1.23 ± 0.26; 95% CI of difference: 0.5 to 1.97; *p* = 0.0097). (**B**) Cystine levels of the Cys-uKPC #7 LV_CTNS-3HA (2.34 ± 1.36 nmol cystine/mg protein) are significantly lower compared to the eGFP (Cys-uKPC #7 LV_eGFP: 8.50 ± 2.02 nmol cystine/mg protein; difference between means: 6.16 ± 1.41; 95% CI of difference: 2.24 to 10.08; *p* = 0.01) and dATP13A2 (Cys-uKPC #7 LV_dATP13A2: 7.38 ± 1.31 nmol cystine/mg protein; difference between means: 5.04 ± 1.09; 95% CI of difference: 2.01 to 8.07; *p* = 0.009) vehicle controls.

Then, in separate transduction experiments per clone, two Cys-uKPC clonal cell lines were complemented with *CTNS* cDNA via LV transduction, followed by antibiotic selection (Cys-uKPC #1 LV_CTNS-3HA; Cys-uKPC #7 LV_CTNS-3HA). As the vehicle control, LV expressing a control protein (eGFP or dATP13A2, an inactivated lysosomal protein—LV_eGFP or LV_dATP13A2) was taken along. Protein expression of *CTNS* cDNA was confirmed by Western blot in both *CTNS* complemented clones (Cys-uKPC #1 LV_CTNS-3HA; Cys-uKPC #7 LV_CTNS-3HA) (Figure S8). In each Cys-uKPC clonal cell line, CTNS protein addition resulted in a significant, about 2.5 to 3-fold, reduction in cystine levels, compared to their vehicle controls (Figure 5; panel A: Cys-uKPC #1; panel B: Cys-uKPC #2).

## 4. Discussion

In this study, we demonstrated that, among the large number of cells voided in the urine of patients with nephropathic cystinosis, undifferentiated cells are present. These cells can be isolated and clonally expanded in vitro, amongst which specific clones express several markers reminiscent of the early stages of nephrogenesis, show a high proliferative capacity and harbor the potential to differentiate towards functional podocytes or PTECs. Therefore, the latter cells can be coined as cystinosis urine-derived kidney progenitor cells (Cys-uKPCs). In contrast with cystinosis patients, in healthy control subjects, no undifferentiated cells are present in urine. This observation can be explained by a potential attempt of cystinosis kidneys to regenerate in compensation for the epithelial cell losses. However, this attempt at regeneration obviously fails, given the progressive deterioration of the renal Fanconi syndrome and chronic kidney disease that ensues.

In our study, we established seven Cys-uKPC clones from the two cystinosis patients that showed the highest yield of clonal colonies growing from their urine in the progenitor medium. One patient had the infantile phenotype and another the juvenile phenotype; these two patients represented the full spectrum of nephropathic cystinosis. Confirming their nature as nephron progenitors, Cys-uKPC expressed *CITED1*, which is a specific marker of the cap mesenchyme cells [48], and *NCAM1*, which has shown to define a population of kidney epithelial cells with clonogenic and stem/progenitor cell properties [14,15]. Not surprisingly, the shortest doubling times and highest proliferative potential were observed in the Cys-uKPC clone with the highest level of co-expression of *NCAM1*, *CITED1*, *VIM* and *PAX2*. However, mRNA expression levels of *CITED1, NCAM1, VIM* and *PAX2*, were lower in Cys-uKPCs compared to nKSPCs, which show a higher potency as kidney progenitor cells as demonstrated by their higher proliferative potential [17]. Cys-uKPCs showed the potential to differentiate between functional podocytes and/or proximal tubular epithelial cells, as demonstrated by the results of gene and protein expression experiments of specific markers and functional assays.

In contrast to previous studies, the kidney progenitor cells of cystinosis patients that we describe here are derived from urine, and the group of undifferentiated cells of which they have been derived has been quantified via a novel accurate method [15,17]. The factors determining the yield of Cys-uKPC that can be isolated from a random urine sample, remain however unclear since no correlation has been observed between the number of undifferentiated cells in the urine and some clinical features of the patients, including the age, cystinosis genotype, the WBC cystine level, eGFR and proteinuria (Figure S3). Nevertheless, Cys-uKPCs show the advantage of a high potential for self-renewal and our approach eliminates the need for laborious isolation and immortalization procedures, while it avoids the complex differentiation protocols from iPSCs, side effects of iPSC reprogramming and the limitations of application of iPSC-derived cell lines [23].

Various hypotheses have been proposed to determine the in vivo origin of regenerating cells of the kidney, depending on their potency and fate [8,9,12,13,15,26]. While an attempt was made to delineate this niche of progenitor cells in vivo, detailed localization of cells in cystinosis kidneys was hampered by a very limited amount of available kidney tissue, as a biopsy is not required for the diagnosis or the clinical follow-up of cystinosis patients. Being able to examine only a single historic cystinosis kidney specimen, we demonstrated cells co-expressing CD133^+^/PAX2^+^ scattered through the tubular epithelium and the parietal epithelium of the Bowman’s capsule. However, no sufficient material was available to perform co-localization studies with specific nephron segment markers. Although we have no direct proof that Cys-uKPCs and CD133^+^/PAX2^+^ cells found in this single cystinosis kidney represent the same cell population, both findings are in line with our hypothesis that epithelial cell loss in the urine of cystinosis patients is accompanied by the presence of cells with nephron progenitor characteristics.

The most intriguing question launched by this study is whether the niche of KPCs found in cystinosis is the response to kidney damage or the reflection of regeneration, which limits the disease progression. Gaide Chevronnay et al. showed in kidney tissue of the cystinosis mouse model that the development of progressive proximal tubular atrophy close to glomerular junctions caused by increased apoptosis, was compensated by a proliferation of PTECs, which was interpreted as an adaptive mechanism of ongoing tissue repair [49,50]. On the other hand, Festa et al. found that abnormal proximal tubule dedifferentiation is part of the pathologic process, resulting in a reduced expression of the PTEC transporters in the apical membrane, resulting in renal Fanconi syndrome [51]. Hence, Cys-KPCs might present the yin-yang endeavor of the cystinosis kidney to regenerate at the expense of proper proximal tubular function.

Another question that has been addressed in our study is whether the pathologic phenotype of Cys-uKPCs can be corrected using a gene addition strategy. Upon complementation of *CTNS*-depleted Cys-uKPCs via LV vector technology, we demonstrated a significant reduction in cystine levels in Cys-uKPCs, indicating that our approach results in a substantial improvement of lysosomal cystine-transporting functions in the *CTNS* complemented cells.

The most practical future application of the Cys-uKPCs described in this study is a novel platform for disease modeling, including the development of organoids and tubuloids. Indeed, urine can serve as a non-invasive, cost-effective and virtually unlimited source for cell lines in cystinosis patients [52,53,54].

Investigating the therapeutic application of gene-corrected Cys-KPCs was beyond the scope of this study. Nevertheless, we might speculate that in vivo complementation of *CTNS* cDNA in the kidney progenitor cell niche in situ, using directly in vivo gene addition with, for example, AAV based viral vectors, could yield promising results [55,56]. For example, in C57BL/6 mice, nephron segment-specific gene expression was shown to be feasible by administration of an AAV9 vector with segment-specific promotors via retrograde ureteric infusion [55]. In another study, specific and highly efficient transduction of kidney stromal and mesangial cells with a synthetic AAV allowing inducible knockout of genes was demonstrated in mice, resulting in a reduction in interstitial fibrosis [56]. These studies might be particularly useful for cystinosis since they highlight a potential route for administration and show the feasibility of targeting renal cells of interest. On the other hand, gene-corrected cystinosis progenitor cells can represent an interesting source of cells for autologous cell therapy. Recently, ex vivo hematopoietic stem cell gene therapy using autologous hematopoietic stem and progenitor cells (HSPCs) transduced with lentiviral vector technology was reported to be successful in Hurler disease, another rare lysosomal storage disorder, yielding extensive metabolic correction in peripheral tissues and the central nervous system [57]. Further studies investigating the potential of Cys-uKPCs to integrate into cystinosis structures in vitro (e.g., organoids) or in (damaged) kidneys in cystinosis animal models, are required to explore these exciting possibilities.

Taken together, we demonstrate that cystinosis patients shed KPCs in urine. These cells can be readily isolated and carry the potential to differentiate between functional podocytes or proximal tubular cells in vitro. We showed that the improvement of the cellular cystine accumulation in cystinosis KPCs can be achieved by ex vivo gene addition, making them a valuable novel in vitro model, and a potential tool for gene therapeutic applications.

## Figures and Tables

**Table 1:** Clinical characteristics of the cystinosis patients included in the study at the moment of culturing urine samples in progenitor medium.

Patient	Age	Sex	Cystinosis Genotype	eGFR	Urine Protein/Creatinine	WBC Cystine	# Clonal Colonies	Cys-uKPC Clones
1	10	F	57kb del + c.926dupG	78	1.97	0.5	0	
2	4	F	Hom 57kb del	>90	2.85	4.26	2	
3	16	M	Hom 57kb del	89	1.82	0.3	9	
4	13	F	c.198_218del + c.926dupG	48	3.04	0.2	16	
5	6	M	57kb del + c.926dupG	>90	4.93	0.32	5	
6 *	13	F	57kb del + c.198_218del	88	1.76	2.45	51	#1–5
7	14	F	Hom 57kb del	53	0.65	2.52	3	
8	4	M	57kb del + IVS 10-7G>A	>90	0.41	1.8	0	
9 *	16	M	Hom 57kb del	46	2.94	3.14	20	#6, #7

Age: expressed in years; eGFR: estimated glomerular filtration rate, expressed as ml/min/1.73 m^2^; urine protein/creatine ratio is expressed as g/g creatinine; WBC: white blood cell; WBC cystine concentration is expressed as nmol ½ cystine/mg protein; #: number; # clonal colonies: number of clonal colonies growing from a single fresh urine sample upon incubation in progenitor medium. *: patients that gave rise to the established Cys-uKPC clones described in this study.

## Data Availability

Not applicable.

## Supplementary Materials

The following supporting information can be downloaded at: https://www.mdpi.com/article/10.3390/cells11071245/s1, Table S1: Control healthy subjects recruited in the study; Table S2: Clinical characteristics of the cystinosis patients whose native kidney specimen was analyzed; Table S3: Antibodies used in Western blot, IF & ICH stainings; Table S4: Primers used to test steady-state mRNA level via qPCR; Figure S1: Protocol of the quantification of undifferentiated cells in urine of cystinosis patients compared with healthy control subjects. Panel A: Establishment of a calibration curve of known numbers of vimentin-positive renal papilla cells. Known numbers of renal papilla cells (1, 5, 10, 50, 100, 300, 500, 1000 cells per well) were seeded using fluorescence activated cell sorting (FACS) in a 96-well plate. cDNA was synthesized and pre-amplified according to the protocol of Picelli et al. qPCR was performed and the resulting Ct value for Beta-actin and vimentin for each known number of renal papilla cells, was used to establish a calibration curve for the total number of cells voided in urine (Ct value for Beta-actin), and the number of undifferentiated cells voided in urine, respectively (Ct value for vimentin). Panel B: Quantification of number of undifferentiated cells in urine of a cystinosis patient or healthy control subject (progenituria). A freshly voided urine sample was collected and a cell pellet was acquired via centrifugation. cDNA was synthesized and pre-amplified according to the protocol of Picelli et al. qPCR was performed and the resulting Ct value for Beta-actin and vimentin for each urine sample was plotted against the established calibration curve for defining the total number of cells voided in urine and the number of undifferentiated cells; Figure S2: Protocol of the isolation, characterization and establishment of Cys-uKPC clones, Cys-uKPC-PTEC and Cys-uKPC-Podo from urine of cystinosis patients; Figure S3: Correlation between the number of undifferentiated cells voided in urine of cystinosis patients, and age, proteinuria (protein/creatinine ratio; g/g creatinine), kidney function (eGFR; mL/min/1.73 m^2^), white blood cell cystine level (WBC cystine level; nmol ½ cystine/mg protein) and cystinosis genotype at the moment of quantification; Figure S4: Immunofluorescence staining for synaptopodin (SYNPO) and podocalyxin (PODXL) in Cys-uKPC-Podocytes. Immunofluorescence pictures of individual color channels (Synaptopodin or Podocalyxin: red; phalloidin: green; DAPI: blue) are provided in order to better discriminate the individual signals. Figure S5: Control stainings for CD133 and PAX2 as markers of kidney progenitor cells; Figure S6: A kidney progenitor cell niche expressing CD133^+^/PAX2^+^ is present in situ in cystinosis kidney; Figure S7: Graphical representation and validation of the SIN lentiviral vector (LV) constructs used in the transduction experiments for complementation of *CTNS* cDNA. Figure S8: Confirmation of protein expression of CTNS-3HA in Cys-uKPC #1 LV_CTNS-3HA and Cys-uKPC #7 LV_CTNS-3HA.
