# Supplementary material for: Urine-Derived Kidney Progenitor Cells in Cystinosis
Source: Cells. 2022 Apr 6;11(7):1245. doi: 10.3390/cells11071245 (PMC8997687; doi:10.3390/cells11071245)

SUPPLEMENTARY MATERIALS

TABLE OF CONTENTS

Supplementary Table S1: Control healthy subjects recruited in the study

Supplementary Table S2: Clinical characteristics of the cystinosis patient whose native kidney specimen was analyzed.

Supplementary Table S3: Antibodies used in western blot, IF & ICH stains.

Supplementary Table S4: Primers used to test steady-state mRNA level via qPCR.

Supplementary Figure S1: Protocol of the quantification of undifferentiated cells in urine of cystinosis patients compared with healthy control subjects.

Supplementary Figure S2: Protocol of the isolation, characterization and establishment of Cys-uKPC clones, Cys-uKPC-PTEC and Cys-uKPC-Podo from urine of cystinosis patients.

Supplementary Figure S3: Correlation between the number of undifferentiated cells voided in urine of cystinosis patients and age, proteinuria (protein/creatinine ratio), kidney function (eGFR), white blood cell cystine level (WBC cystine; nmol ½ cystine/mg protein) and cystinosis genotype at the moment of quantification.

Supplementary Figure S4: Immunofluorescence staining for synaptopodin (SYNPO) and podocalyxin (PODXL) in Cys-uKPC-Podocytes. Immunofluorescence pictures of individual color channels (Synaptopodin or Podocalyxin: red; Phalloidin: green; DAPI: blue) are provided in order to better discriminate the individual signals.

Supplementary Figure S5: Control stainings for CD133 and PAX2 as markers of kidney progenitor cells.

Supplementary Figure S6: A kidney progenitor cell niche expressing $CD133^{+}/PAX2^{+}$ is present *in situ* in cystinosis kidney

Supplementary Figure S7: Graphical representation and validation of the SIN lentiviral vector constructs used in the transduction experiments for complementation of *CTNS* cDNA.

Supplementary Figure S8: Confirmation of protein expression of CTNS-3HA in Cys-uKPC #1 LV_CTNS-3HA and Cys-uKPC #7 LV_CTNS-3HA.

Supplementary Table S1: Control healthy subjects recruited in the study

**Control**	**Age** (years)	**Sex**	**Urine protein/creatinine** (g/g creatinine)
1	8	F	0.23
2	5	F	0.14
3	5	M	0.2
4	10	M	0.1
5	7	F	0.09
6	8	F	0.14
7	4	F	0.19
8	6	F	0.11
9	12	F	0.1

Supplementary Table S2: Clinical characteristics of the cystinosis patient whose native kidney specimen was analyzed.

Sex	M
Genetic background	Hom c992Gins
Age at start hemodialysis (yo)	8
Age at kidney transplantation (yo)	8
Source of kidney specimen	Nephrectomy of native kidney 2 months after kidney transplantation
Indication for nephrectomy	Persistent renal Fanconi syndrome after transplantation
Proteinuria prior to nephrectomy (mg/l)	830

Supplementary Table S3: Antibodies used in western blot, IF & ICH stainings

Name	Target	Manufacturer	Catalog #	Dilution
**Immunofluorescence staining**				
Primary antibodies				
mouse anti-synaptopodin	SYNPO	Progen Biotechnik	65294	1:100
rabbit anti-podocalyxin (C-terminal)	PODXL	Abcam®	Ab154305	1:1000
mouse anti-HA.11 epitope tag	HA.11	BioLegend®	901515	1:1000
rabbit anti-LAMP-1	LAMP-1	Cell Signaling Tech	#9091	1:200
Secondary antibodies				
donkey anti-mouse IgG H&L Alexa Fluor® 555		Abcam®	Ab150106	1:300
goat anti-rabbit IgG Alexa Fluor® 546		Invitrogen™ Thermo Fisher	A11035	1:400
**Western Blot**				
Primary antibodies				
rabbit anti-aquaporin 1	AQP1	Novus Biologicals	NBP1-84488	1:1000
mouse anti-synaptopodin	SYNPO	Santa Cruz	Sc-515842	1:500
rabbit anti-podocalyxin	PODXL	Abcam®	Ab154305	1:500-1:1000
mouse anti-Beta Actin	ACTB	Merck Sigma	A5441	1:10.000
rabbit anti-Beta Actin	ACTB	Cell Signaling Tech	4970S	1:1000
rabbit anti-GAPDH	GAPDH	Abcam®	Ab9485	1:2000
Secondary antibodies				
goat anti-rabbit IgG horseradish peroxidase-conjugated (HRP)		Agilent Dako	P0448	1:2000-1:10.000
goat anti-mouse IgG horseradish peroxidase-conjugated (HRP)		Dako	P0447	1:2000
**Immunohistochemistry**				
Primary antibodies				
rabbit anti-PAX2	PAX2	Abcam®	Ab79389	1:250
mouse anti-CD133	CD133	Thermo Fisher	MA1-219	1:100
Secondary antibodies				
goat anti-rabbit IgG (H+L) Alexa Fluor® 546		Invitrogen™	A11035	1:400-1:500
goat anti-mouse IgG H&L Alexa Fluor® 488		Abcam®	Ab150113	1:300-1:500

Supplementary Table S4: Primers used to test steady-state mRNA level via qPCR

Target gene	Gene ID	Protein	Forward primer (5′-3′)	Reverse primer (5′-3′)
ACTB	60	β-Actin	AAGAGCTACGAGCTGCCTGA	GACTCCATGCCCAGGAAGG
CTNS	1497	cystinosin	CCACAGGCCTACATGAACTT	TCCACTGGTCGTTGTTGTAG
**Kidney stem/progenitor cell markers**				
NCAM1	4684	Neural cell adhesion molecule 1	GTCCTGCTCCTGGTGGTTGT	TGACCGCAATGCACATGAA
CITED1	4435	Cbp/p300 interacting transactivator with Glu/Asp rich carboxy-terminal domain 1	AGGATGCCAACCAAGAGATG	TGGTTCCATTTGAGGCTACC
VIM	7431	vimentin	ACACCCTGCAATCTTTCAGACA	GATTCCACTTTGCGTTCAAGGT
PAX2	5076	paired box 2	AACGACAGAACCCGACTATG	ATCCCACTGGGTCATTGGAG
**Proximal tubular epithelial cell markers**				
ABCB1	5243	ATP binding cassette subfamily B member 1; MDR-1; P-gp	AGCTTAACACCCGACTTACAGA	ACCTCTTCAGCTACTGCTCCAGCT
AQP1	358	Aquaporin 1	ATCGAGATCATCGGGACCCTCCA	TGTCGTCGGCATCCAGGTCATA
**Podocyte markers**				
SYNPO	11346	synaptopodin	AGCCCAAGGTGACCCCGAAT	CCCTGTCACGAGGTGCTGGC
PODXL	5420	podocalyxin	CTTGAGACACAGACACAGAG	CCGTATGCCGCACTTATC
WT1	7490	Wilm's tumor protein 1	GGACAGAAGGGCAGAGCAACCA	GTCTCAGATGCCGACCGTACAA

Supplementary Figure S1: Protocol of the quantification of undifferentiated cells in urine of cystinosis patients compared with healthy control subjects. **Panel A:** Establishment of a calibration curve of known numbers of vimentin-positive renal papilla cells. Known numbers of renal papilla cells (1, 5, 10, 50, 100, 300, 500, 1000 cells per well) were seeded using fluorescence activated cell sorting (FACS) in a 96-well plate. cDNA was synthesized and pre-amplified according to the protocol of Picelli et al. qPCR was performed and the resulting Ct value for Beta-actin and vimentin for each known number of renal papilla cells, was used to establish a calibration curve for the total number of cells voided in urine (Ct value for Beta-actin), and the number of undifferentiated cells voidedin urine respectively (Ct value for vimentin). **Panel B:** Quantification of number of undifferentiated cells in urine of a cystinosis patient or healthy control subject ('progenituria'). A freshly voided urine sample was collected and a cell pellet acquired via centrifugation. cDNA was synthesized and pre-amplified according to the protocol of Picelli et al. qPCR was performed and the resulting Ct value for Beta-actin and vimentin for each urine sample, was plotted against the established calibration curve for defining the total number of cells voided in urine and the number of undifferentiated cells.

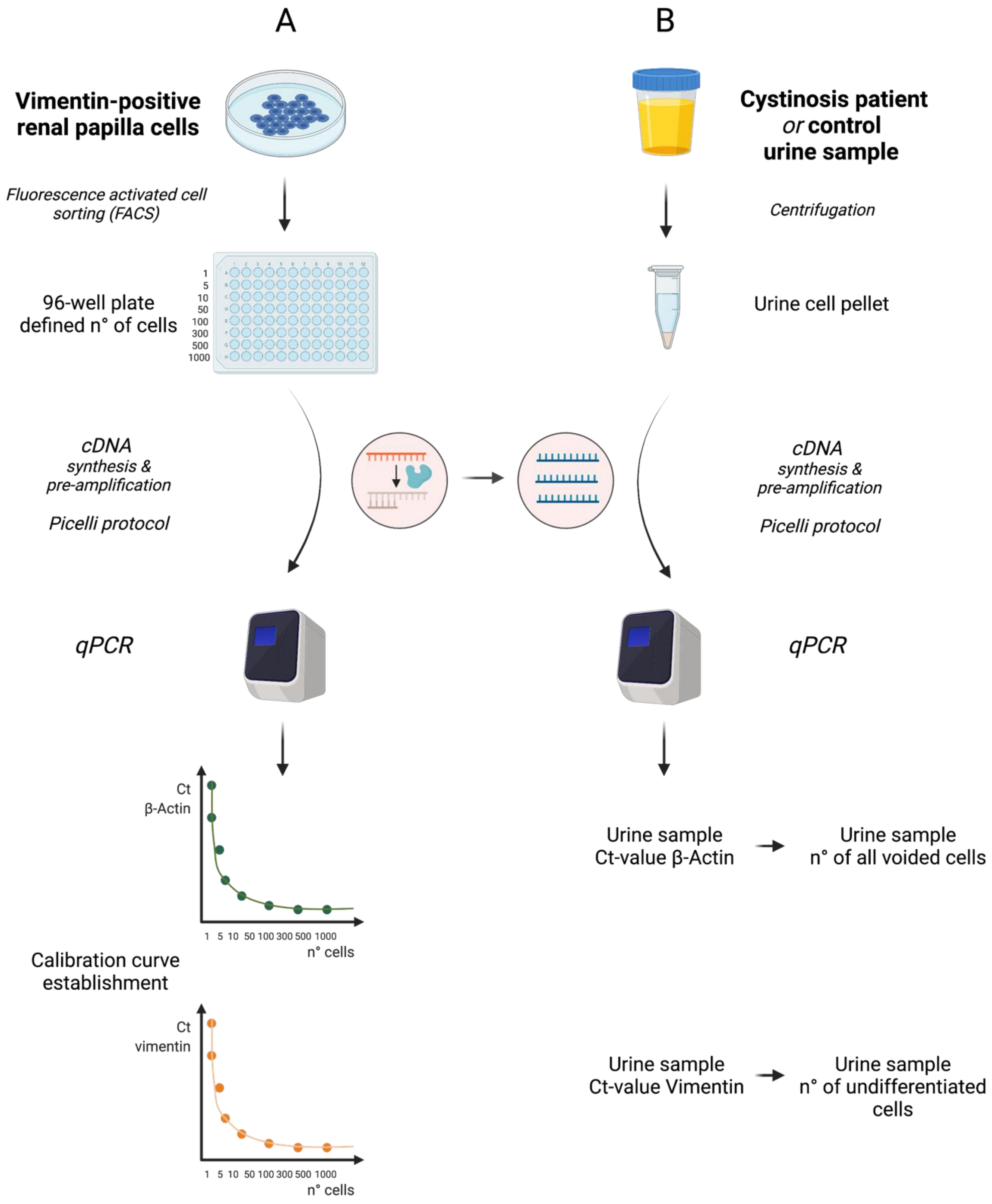
A
B
Vimentin-positive renal papilla cells
Fluorescence activated cell sorting (FACS)
96-well plate defined n° of cells
1
5
10
50
100
300
500
1000
cDNA synthesis & pre-amplification
Picelli protocol
qPCR
Ct β-Actin
1 5 10 50 100 300 500 1000
n° cells
Calibration curve establishment
Ct vimentin
1 5 10 50 100 300 500 1000
n° cells
Cystinosis patient or control urine sample
Centrifugation
Urine cell pellet
cDNA synthesis & pre-amplification
Picelli protocol
qPCR
Urine sample Ct-value β-Actin
Urine sample n° of all voided cells
Urine sample Ct-value Vimentin
Urine sample n° of undifferentiated cells

Supplementary Figure S2: Protocol of the isolation, characterization and establishment of Cys-uKPC clones, Cys-uKPC-PTEC and Cys-uKPC-Podo from urine of cystinosis patients.

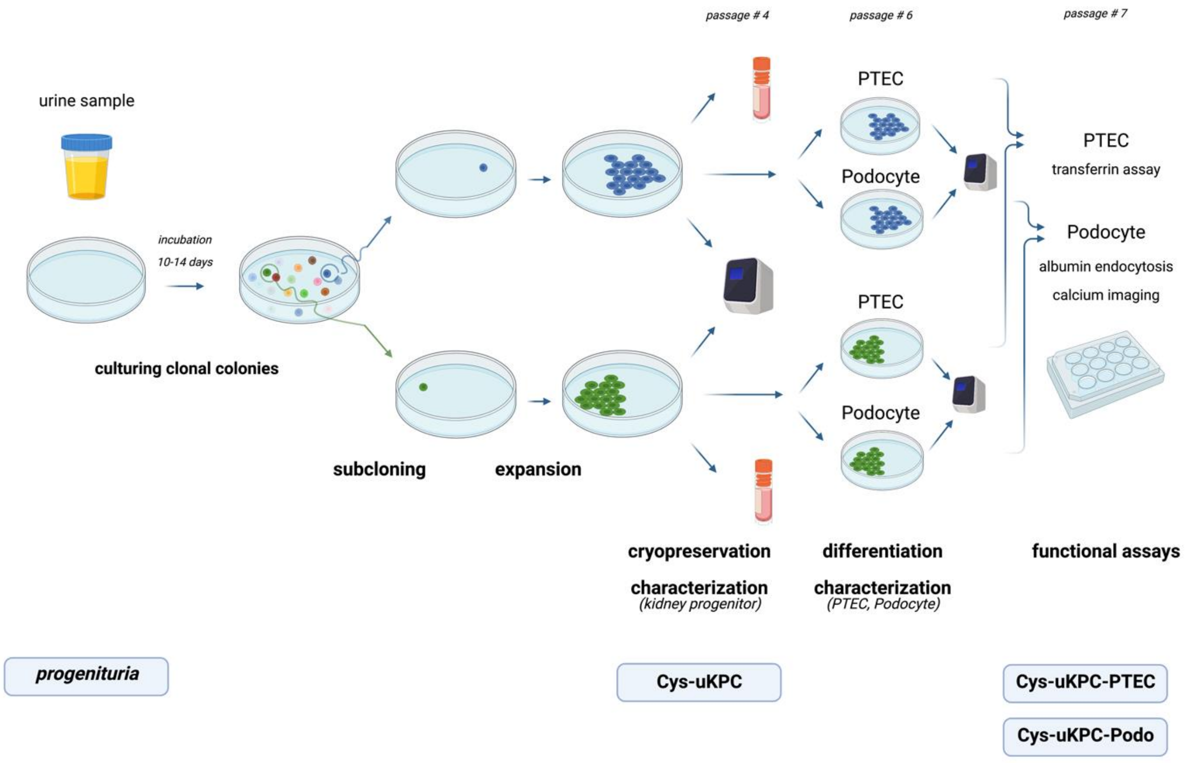

Supplementary Figure S3: Correlation between the number of undifferentiated cells voided in urine of cystinosis patients, and age, proteinuria (protein/creatinine ratio; g/g creatinine), kidney function (eGFR; ml/min/1.73m^2), white blood cell cystine level (WBC cystine; nmol ½ cystine/mg protein) and cystinosis genotype at the moment of quantification. No correlation could be demonstrated with any parameter.

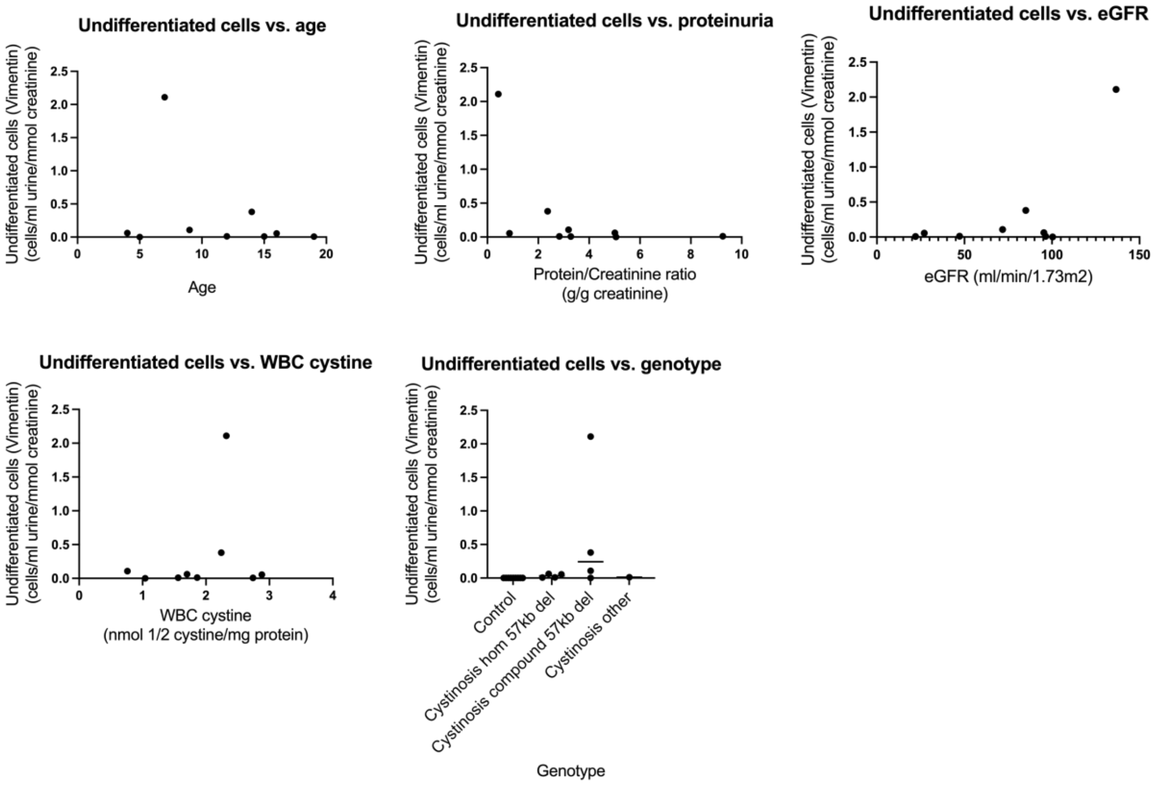

Supplementary Figure S4: Immunofluorescence staining for synaptopodin (SYNPO) and podocalyxin (PODXL) in Cys-uKPC-Podocytes. Immunofluorescence pictures of individual color channels (Synaptopodin or Podocalyxin: red; Phalloidin: green; DAPI: blue) are provided in order to better discriminate the individual signals.

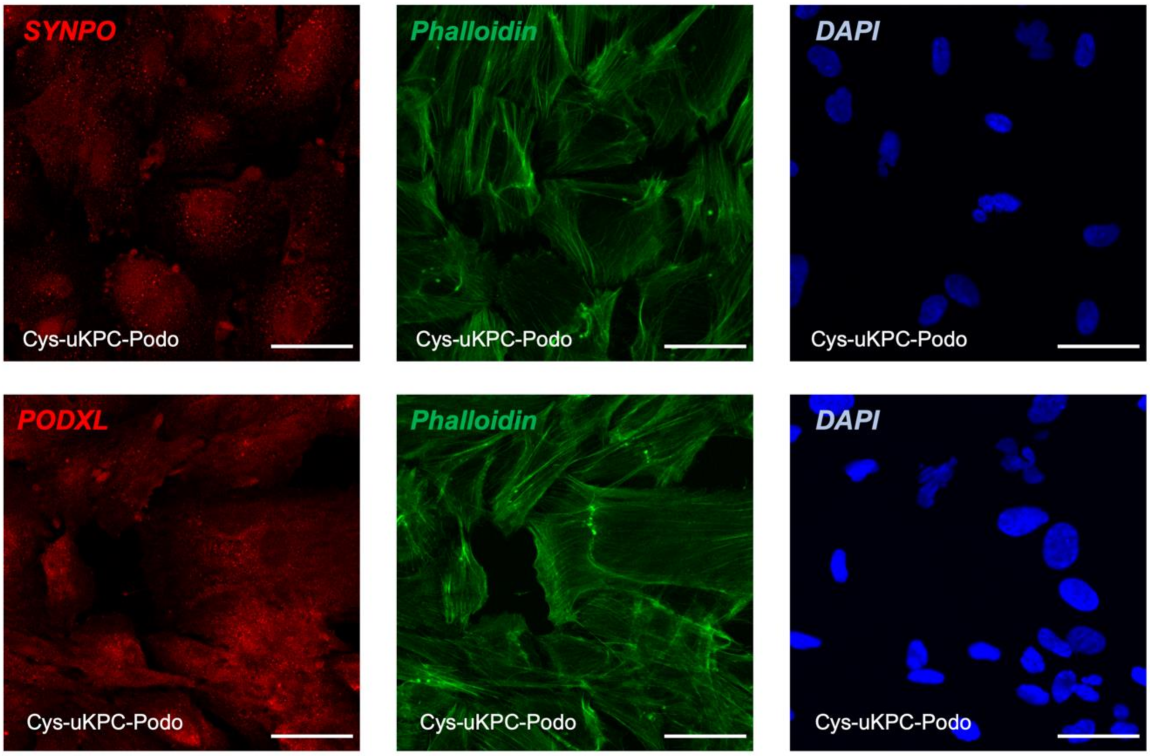

Supplementary Figure S5: Control stainings for CD133 and PAX2 as markers of kidney progenitor cells. **Panel A & B:** Positive control for immunohistochemistry (A) and immunofluorescence staining (B) for PAX2 in healthy fetal kidney tissue (28 weeks gestational age) demonstrates a strong presence of PAX2 at the superficial nephrogenic rim, in contrast to regions of differentiated cells. Scale bar panel A: 200 µm; scale bar panel B: 50 µm.

Panel C & D: Positive control immunohistochemistry for CD133 of an adult transplant kidney in a 13-year old recipient, demonstrating cytoplasmic positivity in epithelial cells of the Bowman's capsule and tubular epithelial cells, of which in some the apical border is strongly positive. Scale bar panel C: 25 µm; scale bar panel D: 50 µm. **Panel E:** Positive control for immunofluorescence staining for CD133 in the context of acute tubular injury, showing positivity at the apical border of tubular epithelial cells. Scale bar: 50 µm. **Panel F:** Control immunofluorescence co-staining for CD133 and PAX2 in a kidney tissue specimen of minimal changes disease-type nephrotic syndrome shows no signal for either marker. Scale bar: 50 µm.

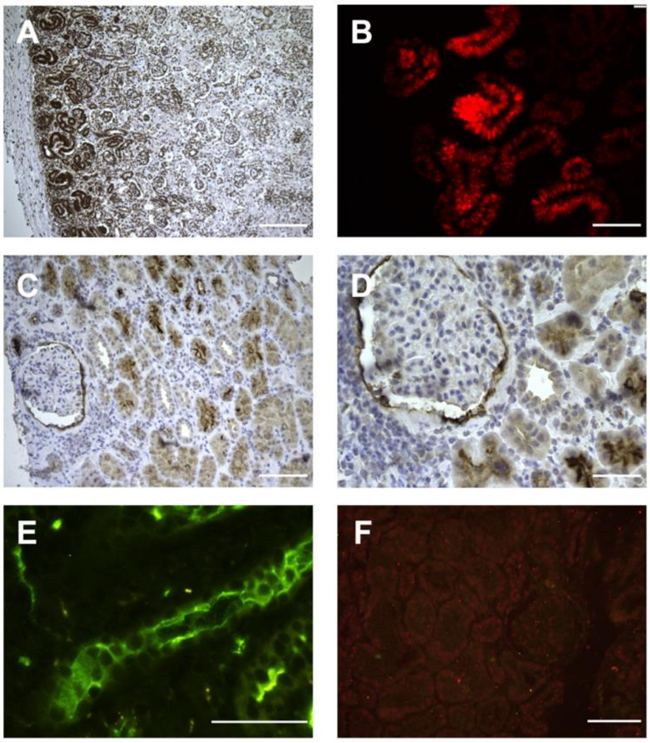

Supplementary Figure S6: A kidney progenitor cell niche expressing CD133[+]/PAX2[+] is present *in situ* in cystinosis kidney

Panel A & B: Immunofluorescence co-staining for CD133 (green) and PAX2 (red) in tissue specimens of the native kidney of a nephropathic cystinosis patient, showing focal co-expression in cells scattered throughout the tubular epithelium (full white arrows in panel A, B) and the parietal epithelial cells of the Bowman's capsule (dashed white arrows in panel A). Scale bar: 50 µm.

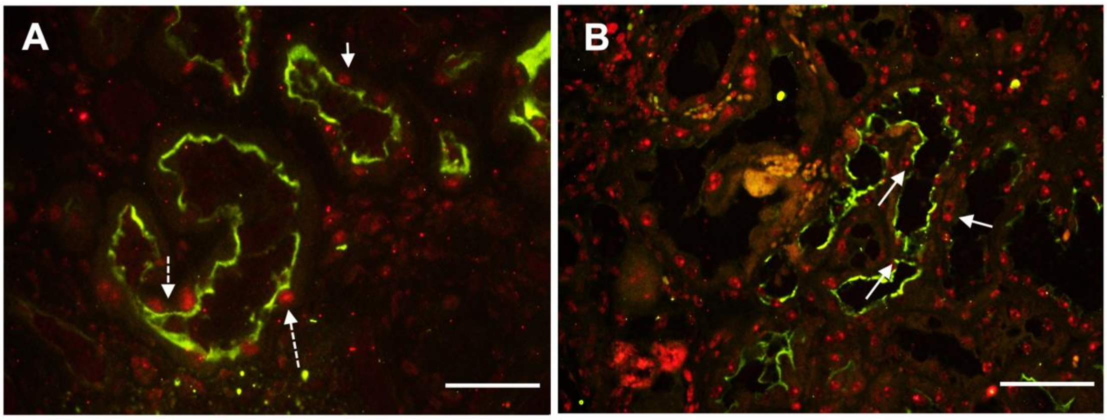

Supplementary Figure S7: Graphical representation and validation of the SIN lentiviral vector (LV) constructs used in the transduction experiments for complementation of *CTNS*.

Panel A: Graphical representation of the second-generation SIN lentiviral vector transfer plasmid construct used to generate viral vectors for the transduction experiments to complement Cys-uKPCs with *CTNS* cDNA or *eGFP* as control. SIN-lentiviral vector expressing 3HA-tagged *CTNS* cDNA driven from an EF1a promoter, together with a puromycin resistance selection cassette. **Panel B:** Validation of CTNS-3HA expressing LVs; detection of the 3HA tag (green) in fibroblasts expressing the CTNS-3HA construct upon transduction with the CTNS-3HA LV. **Panel C:** LAMP1 staining (red) visualizing the late-endosomal/lysosomal compartements. **Panel D:** Merge of the 3HA signal with the LAMP1 signal indicating that the punctated signal of the CTNS-3HA correspends to a lysosomal staining pattern. **Panel E:** WB detecting the CTNS-3HA or vinculin (equal loading) upon transduction of fibroblasts. Migration pattern of CTNS-3HA correspond to the glycosylated version of the CTNS protein. NT = Not transduced; LV = lentiviral vector.

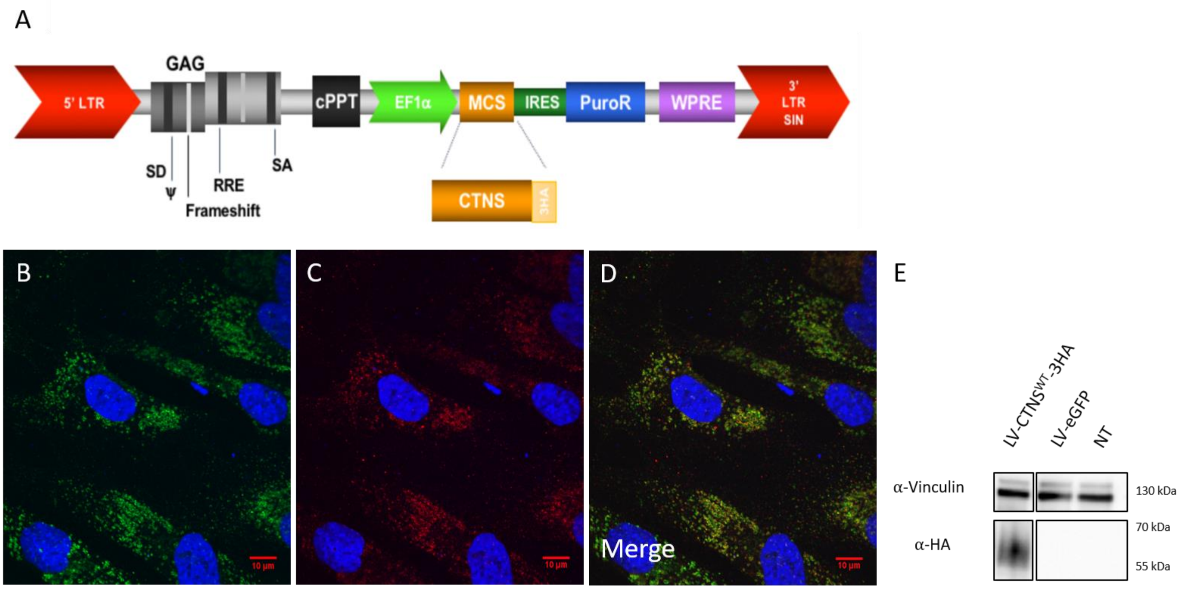
A
GAG
5' LTR
cPPT
EF1α
MCS
IRES
PuroR
WPRE
3' LTR SIN
SD
ψ
RRE
Frameshift
SA
CTNS
3HA
B
C
D
Merge
E
LV-CTNSWT-3HA
LV-eGFP
NT
α-Vinculin
α-HA
130 kDa
70 kDa
55 kDa

Supplementary Figure S8: Confirmation of protein expression of CTNS-3HA in Cys-uKPC #1 LV_CTNS-3HA and Cys-uKPC #7 LV_CTNS-3HA. Western Blot for 3HA-tagged CTNS in the stable CTNS-3HA expressing Cys-uKPC #1 LV_CTNS-3HA (panel A) and Cys-uKPC #7 LV_CTNS-3HA (panel B), compared to GAPDH as housekeeping protein, and the eGFP expressing vehicle controls Cys-uKPC #1 LV_eGFP and Cys-uKPC #7 LV_eGFP. Neonatal kidney stem progenitor cells (nKSPC) and the non-transduced Cys-uKPCs (Cys-uKPC #1 and Cys-uKPC #7) were also taken along as controls.

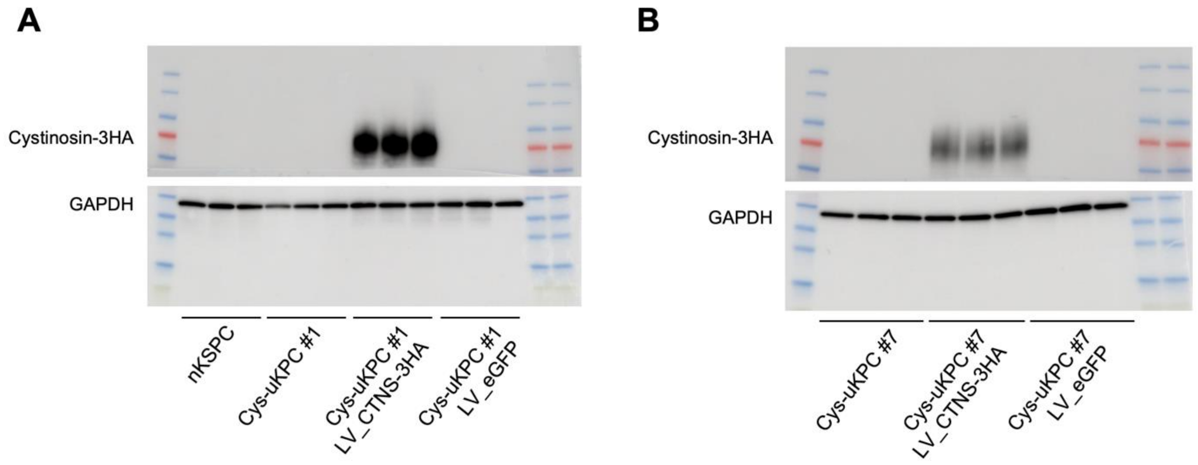

Supplement: Supplementary file 1 [file cells-11-01245-s001.zip › cells-1599823-supplementary.pdf]
